# Age group performances in 100 km and 100 miles ultra-marathons

**DOI:** 10.1186/2193-1801-3-331

**Published:** 2014-07-01

**Authors:** Christoph A Rüst, Thomas Rosemann, Matthias A Zingg, Beat Knechtle

**Affiliations:** Institute of General Practice and Health Services Research, University of Zurich, Zurich, Switzerland; Gesundheitszentrum St. Gallen, Vadianstrasse 26, 9001 St. Gallen, Switzerland

**Keywords:** Running, Ultra-distance, Women, Men

## Abstract

Improved performance has been reported for master runners (*i.e.* athletes older than 40 years) in both single marathons and single ultra-marathons. This study investigated performance trends of age group ultra-marathoners competing in all 100 km and 100 miles races held worldwide between 1971 and 2013. Changes in running speeds across years were investigated for the annual ten fastest 5-year age group finishers using linear, non-linear and multi-level regression analyses. In 100 km, running speed remained unchanged in women in 25–29 years, increased non-linearly in 30–34 to 55–59 years, and linearly in 60–64 years. In men, running speed increased non-linearly in 18–24 to 60–64 years and linearly in 65–69 to 75–79 years. In 100 miles, running speed increased in women linearly in 25–29 and 30–34 years, non-linearly in 35–39 to 45–49 years, and linearly in 50–54 and 55–59 years. For men, running speed increased linearly in 18–24 years, non-linearly in 25–29 to 45–49 years, and linearly in 50–54 to 65–69 years. Overall, the faster race times over the last 30 years are a result of all top ten finishers getting faster. These findings suggest that athletes in younger to middle age groups (*i.e.* 25–35 to 50–65 years depending upon sex and distance) have reached their limits due to a non-linear increase in running speed whereas runners in very young (*i.e.* younger than 25–35 years) and older age groups (*i.e.* older than 50–65 years) depending upon sex and distance might still improve their performance due to a linear increase in running speed.

## Background

During the last decades, an increase in the number of master runners (*i.e.* runners older than 35 years) has been reported for longer running distances such as marathons (Jokl et al. 2004; Lepers and Cattagni 2012) and ultra-marathons (Hoffman 2010; Hoffman et al. 2010a; Hoffman and Wegelin 2009; Knechtle et al. 2012b; Rüst et al. 2013; Zingg et al. 2014). In addition to the increase in the participation in master runners, an improvement in performance for older athletes has been reported for marathoners and ultra-marathoners (Hoffman and Wegelin 2009; Jokl et al. 2004; Knechtle et al. 2012b; Lepers and Cattagni 2012; Reaburn and Dascombe 2009).

In marathon running, Jokl et al. (2004) reported for the ‘New York City Marathon’ between 1983 and 1999 a greater increase in the number of master participants older than 50 years than younger runners. Lepers and Cattagni (2012) showed that the participation of master runners increased to a greater extent for women than for men during the 1980–2009 period at the ‘New York City Marathon’. Additionally, marathon race times of the fastest master runners at the ‘New York City Marathon’ decreased significantly in men > 64 years and in women > 44 years (Lepers and Cattagni 2012). Jokl et al. (2004) reported that master runners at the ‘New York City Marathon’ improved marathon race times to a greater extent compared to younger runners. Women aged 40–69 years showed a significant decrease in marathon race times with the greatest time improvements (*i.e.* 3.79 min/year) in runners in age group 60–69 years. Lower improvements in performance (*i.e.* 2.08 min/year) were reported for women in age group 50–59 years. For men, the highest improvements were found in older age groups. Improvements were highest in runners in age group 70–79 years (*i.e.* 1.9 min/year), followed by runners in age group 60–69 years (*i.e.* 1.23 min/year) and runners in age group 50–59 years (*i.e.* 0.13 min/year).

Ultra-marathons are mainly held as 100 km (Cejka et al. 2014; Knechtle et al. 2012b) and 100 miles (Hoffman 2010; Hoffman et al. 2010b; Hoffman and Wegelin 2009) ultra-marathons. Similarly to the findings for the marathon distance, the percentage of finishers decreased in age groups 18–29 and 30–39 years and increased in age groups 40–49 and 50–59 years in ultra-marathons such as the ‘100 km Lauf Biel’ held in Switzerland (Knechtle et al. 2012b). It has been observed that the number and the mean age of finishers increased for 100 miles ultra-marathons such as the ‘Western States 100-Mile Endurance Run’ in the USA (Hoffman and Wegelin 2009). The increase in the mean age in 100 miles ultra-marathoners was due to a growth in participation of women > 40 years and men > 50 years (Hoffman and Wegelin 2009). Regarding these two examples of ultra-marathon running races, it seemed that the changes in performance across the years were different between 100 km and 100 miles ultra-marathoners. In the ‘100 km Lauf Biel’, running performance of the top ten overall decreased between 1998 and 2010 in men while it remained stable in women (Knechtle et al. 2012b). In contrast, in the ‘Western States 100-Mile Endurance Run’ in the USA, the top five women overall and the top five women in age groups 30–39 years and 40–49 years improved race times over the 1974–2007 period (Hoffman and Wegelin 2009).

The rapid increase of the ultra-running phenomenon worldwide (Freund et al. 2012; International Association of Ultrarunners, http://www.iau-ultramarathon.org; Schütz et al. 2012) is intriguing and it is of interest to examine how elderly runners > 35 years are implicated. A problem of the mentioned studies was the use of linear analyses to investigate a potential change in running performance. A linear change (*i.e.* a linear increase in running speed) would mean that performance would improve infinitely during the next decades and centuries. However, sport as a whole shows generally a non-linear improvement (Reinboud 2004).

Previous studies investigated participation and performance trends in runners and ultra-runners in single races (Jokl et al. 2004; Lepers and Cattagni 2012; Hoffman 2010; Hoffman and Wegelin 2009; Knechtle et al. 2012b), a series of races within a country such as Germany (Leyk et al. 2007), or a race series held within a continent such as North America (Hoffman et al. 2010b). However, it is not known whether the reported trends were restricted to a specific race or a specific area. So far, data of worldwide trends are missing. The purpose of the present study was to investigate performance trends of age group runners competing in 100 km and 100 miles ultra-marathons held worldwide between 1971 and 2013 by using linear and non-linear regression analyses. Based upon previous findings for single races and single race series, we hypothesized also for an analysis of world-wide data from 100 km and 100 miles ultra-marathons an increase in participation of master ultra-runners and an improvement of master athletes’ running performance. We assume that athletes competing in younger age groups have reached their limits (*i.e.* a non-linear increase in running speed across years) whereas athletes competing in older age groups might still be improving their performance (*i.e.* a linear increase in running speed across years).

## Methods

### Ethics

The present study was approved by the Institutional Review Board of St. Gallen, Switzerland, with a waiver of the requirement for informed consent given that the study involved the analysis of publicly available data.

### Data sampling and data analysis

The data set for this study was obtained from the website ‘Deutsche Ultramarathon Vereinigung’ (http://www.ultra-marathon.org). Race times and ages of all women and men who ever finished a 100 km and a 100 miles ultra-marathon worldwide between 1971 and 2013 were collected. This data base records all race results in ultra-marathon races held worldwide. The last author retrieved all data from this data base in http://statistik.d-u-v.org/geteventlist.php by searching for all races held in 100 km and all races held in 100 miles. The second to last author prepared all data for statistical analysis and the first author performed the statistical analyses. To analyse performance trends in the different age groups, women and men were divided into age groups of five years intervals (*i.e.* 18–24, 25–29, 30–34, 35–39, 40–44, 45–49, 50–54, 55–59, 60–64, 65–69, 70–74, and 75–79 years). For both 100 km and 100 miles ultra-marathons, the annual top ten women and men were considered for each age group.

### Statistical analysis

Each set of data was tested for normal distribution and for homogeneity of variances prior to statistical analyses. Normal distribution was tested using a D’Agostino and Pearson omnibus normality test and homogeneity of variances was tested using a Levene’s test. Trends in participation were analysed using regression analysis with ‘straight line’ and ‘exponential growth equation’ model, whereas for each set of data (*e.g.* each age group) both models were compared using Akaike’s Information Criteria (AICc) to decide which of the two models showed the higher probability of correctness. Single and multi-level regression analyses investigated changes in running speed. A hierarchical regression model avoided the impact of a cluster-effect on results when a particular athlete finished more than once in the annual top ten. Regression analyses for running speed were corrected for age of the athletes to prevent a misinterpretation of the ‘age-effect’ as a ‘time-effect’ since age is an important predictor variable in ultra-endurance performance such as 100 km ultra-marathon running (Knechtle et al. 2010a). Since the changes in endurance performance and sex differences in endurance performance are assumed to be non-linear (Reinboud 2004), we additionally calculated the non-linear regression. When the best-fit model was a non-linear (*i.e.* polynomial) regression, we compared the non-linear to the linear model using AIC and F-test in order to show which of the two models would be more appropriate to explain the trend of the data. Statistical analyses were performed using IBM SPSS Statistics (Version 22, IBM SPSS, Chicago, IL, USA), CurveExpert Professional (Version 2.0.3, Hyams D.G.) and GraphPad Prism (Version 6.01, GraphPad Software, La Jolla, CA, USA). Significance was accepted at *P* < 0.05 (two-sided for *t*-tests). Data in the text and figures are given as mean ± standard deviation (SD).

## Results

**Figure 1:**
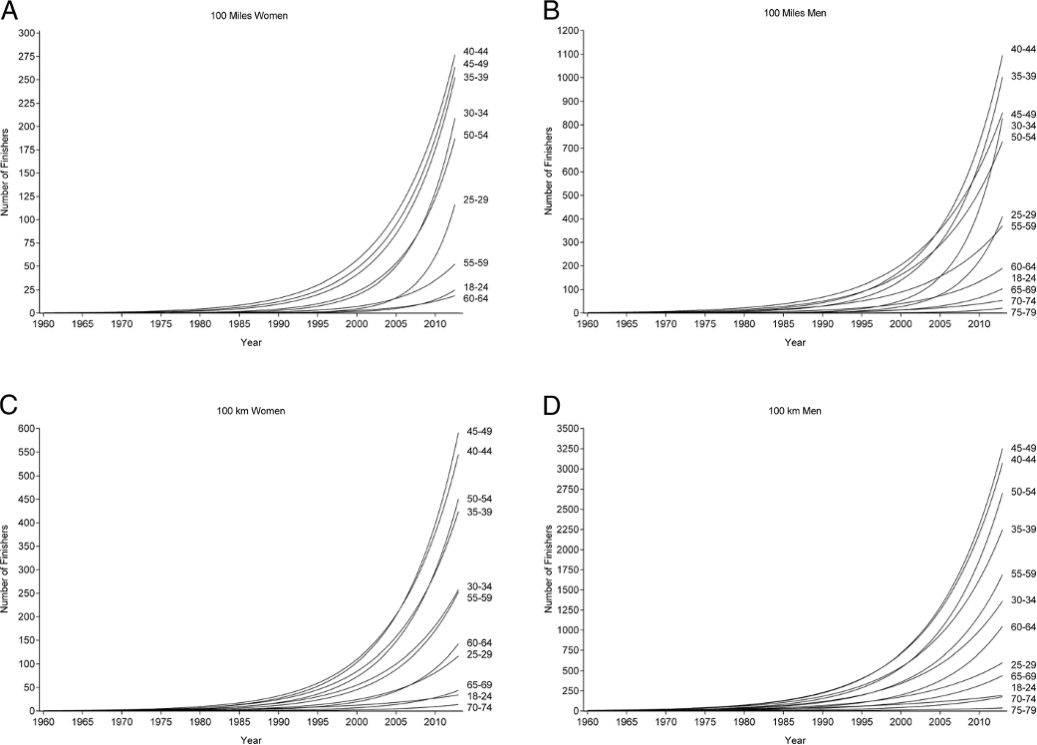
**Trends in finishers.** Women 100 miles **(Panel A)**, men 100 miles **(Panel B)**, women 100 km **(Panel C)**, men 100 km **(Panel D)**.

The number of successful finishers increased exponentially in all age groups in 100 miles races for both women (Figure 1A) and men (Figure 1B) and also in 100 km races for both women (Figure 1C) and men (Figure 1D).

**Figure 2:**
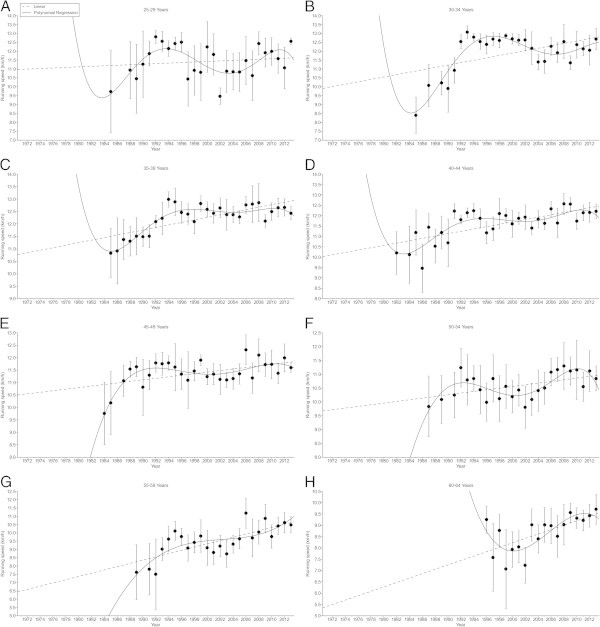
**Running speed in female 100 km age group athletes.** 25-29 years **(Panel A)**, 30-34 years **(Panel B)**, 35-39 years **(Panel C)**, 40-44 years **(Panel D)**, 45-49 years **(Panel E)**, 50-54 years **(Panel F)**, 55-59 years **(Panel G)**, 60-64 years **(Panel H)**.

**Figure 3:**
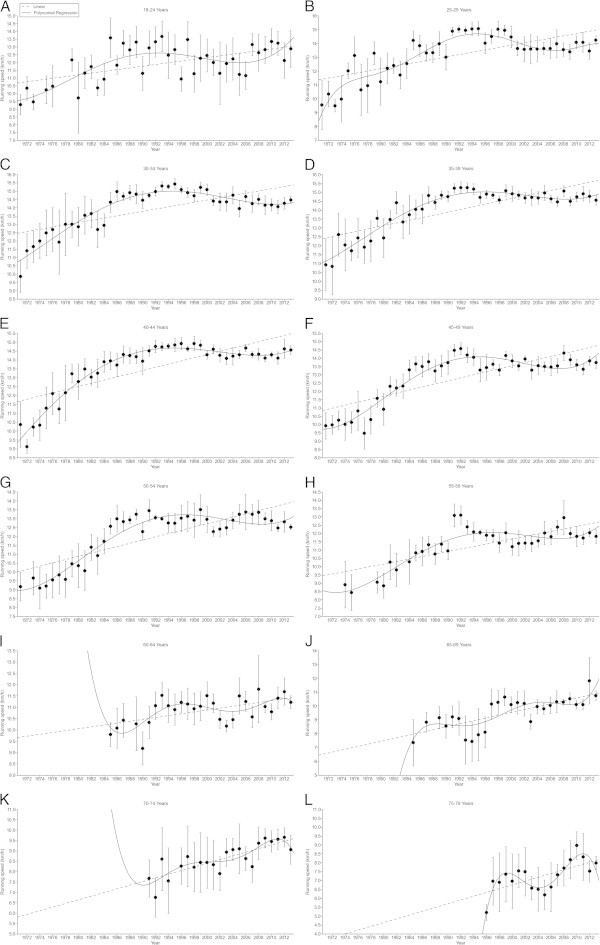
**Running speed in male 100 km age group athletes.** 18-24 years **(Panel A)**, 25-29 years **(Panel B)**, 30-34 years **(Panel C)**, 35-39 years **(Panel D)**, 40-44 years **(Panel E)**, 45-49 years **(Panel F)**, 50-54 years **(Panel G)**, 55-59 years **(Panel H)**, 60-64 years **(Panel I)**, 65-69 years **(Panel J)**, 70-74 years **(Panel K)**, 75-79 years **(Panel L)**.

**Figure 4:**
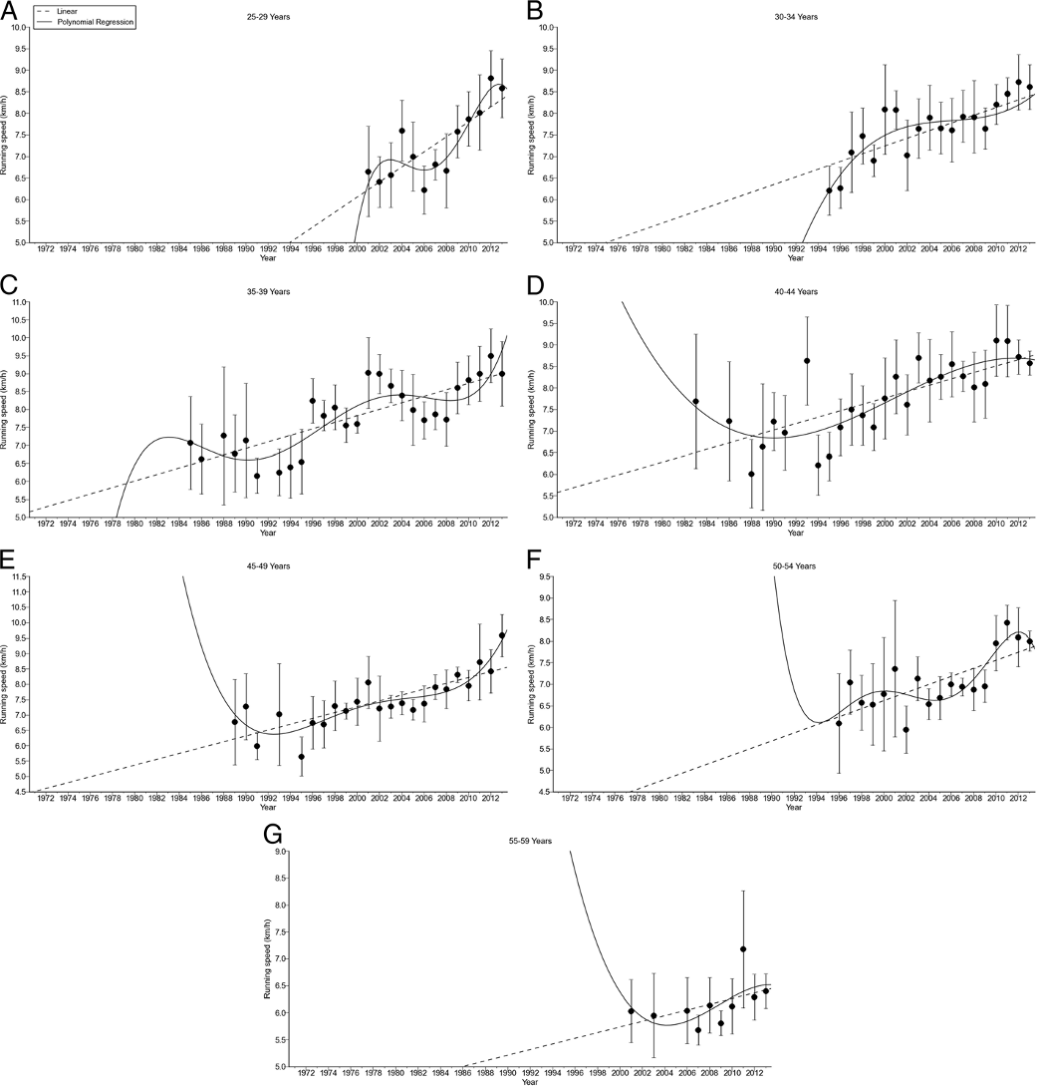
**Running speed in female 100 miles age group athletes.** 25-29 years **(Panel A)**, 30-34 years **(Panel B)**, 35-39 years **(Panel C)**, 40-44 years **(Panel D)**, 45-49 years **(Panel E)**, 50-54 years **(Panel F)**, 55-59 years **(Panel G)**.

**Figure 5:**
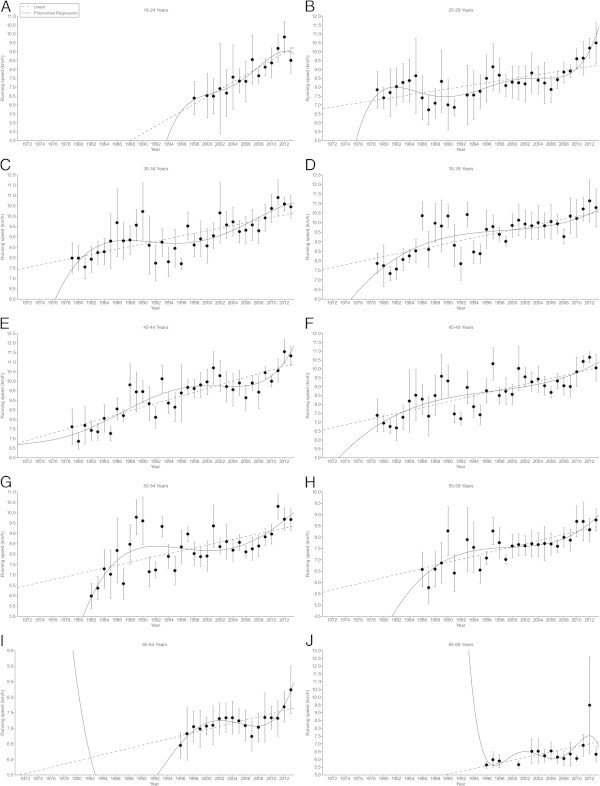
**Running speed in male 100 miles age group athletes.** 18-24 years **(Panel A)**, 25-29 years **(Panel B)**, 30-34 years **(Panel C)**, 35-39 years **(Panel D)**, 40-44 years **(Panel E)**, 45-49 years **(Panel F)**, 50-54 years **(Panel G)**, 55-59 years **(Panel H)**, 60-64 years **(Panel I)**, 65-69 years **(Panel J)**.

Figures 2, 3, 4, 5 present the changes in running speed in 100 km and 100 miles for women and men. Table 1 presents the trends of the changes and Table 2 the absolute running speeds and the percent changes. In 100 km in women, running speed remained unchanged in age group 25–29 (Figure 2A), increased non-linearly in age groups 30–34 (Figure 2B), 35–39 (Figure 2C), 40–44 (Figure 2D), 45–49 (Figure 2E), 50–54 (Figure 2F), and 55–59 years (Figure 2G), but linearly in age group 60–64 years (Figure 2H). The highest percent increase in running speed was found in age group 30–34 years (Table 2). In 100 km in men, running speed increased non-linearly in age groups 18–24 (Figure 3A), 25–29 (Figure 3B), 30–34 (Figure 3C), 35–39 (Figure 3D), 40–44 (Figure 3E), 45–49 (Figure 3F), 50–54 (Figure 3G), 55–59 (Figure 3H), and 60–64 years (Figure 3I), but linearly in age groups 65–69 (Figure 5J), 70–74 (Figure 3K), and 75–79 years (Figure 3L). The highest percent increase occurred in age group 75–79 years (Table 2).

**Table 1:** **Multi-level regression analyses for the changes in running speed of the annual ten fastest female and male age group finishers across years in 100 km and 100 miles after correction for multiple finishes and age of athletes with multiple finishes**

	***ß***	***p***	Trend of the change across years
**100 km women**			
**25–29 years**	0.015	0.167	no change
**30–34 years**	0.070	< 0.001	non-linear polynomial regression 5^th^ degree
**35–39 years**	0.051	< 0.001	non-linear polynomial regression 5^th^ degree
**40–44 years**	0.056	< 0.001	non-linear polynomial regression 5^th^ degree
**45–49 years**	0.031	< 0.001	non-linear polynomial regression 4^th^ degree
**50–54 years**	0.027	< 0.001	non-linear polynomial regression 5^th^ degree
**55–59 years**	0.098	< 0.001	non-linear polynomial regression 3^rd^ degree
**60–64 years**	0.098	< 0.001	linear
**100 km men**			
**18–24 years**	0.056	< 0.001	non-linear polynomial regression 5^th^ degree
**25–29 years**	0.085	< 0.001	non-linear polynomial regression 6^th^ degree
**30–34 years**	0.068	< 0.001	non-linear polynomial regression 4^th^ degree
**35–39 years**	0.077	< 0.001	non-linear polynomial regression 4^th^ degree
**40–44 years**	0.089	< 0.001	non-linear polynomial regression 4^th^ degree
**45–49 years**	0.092	< 0.001	non-linear polynomial regression 4^th^ degree
**50–54 years**	0.092	< 0.001	non-linear polynomial regression 4^th^ degree
**55–59 years**	0.076	< 0.001	non-linear polynomial regression 4^th^ degree
**60–64 years**	0.041	< 0.001	non-linear polynomial regression 4^th^ degree
**65–69 years**	0.104	< 0.001	linear
**70–74 years**	0.088	< 0.001	linear
**75–79 years**	0.107	< 0.001	linear
**100 miles women**			
**25–29 years**	0.175	< 0.001	linear
**30–34 years**	0.090	< 0.001	linear
**35–39 years**	0.091	< 0.001	non-linear polynomial regression 3^rd^ degree
**40–44 years**	0.074	< 0.001	non-linear polynomial regression 3^rd^ degree
**45–49 years**	0.093	< 0.001	non-linear polynomial regression 4^th^ degree
**50–54 years**	0.094	< 0.001	linear
**55–59 years**	0.052	0.005	linear
**100 miles men**			
**18–24 years**	0.199	< 0.001	linear
**25–29 years**	0.057	< 0.001	non-linear polynomial regression 5^th^ degree
**30–34 years**	0.064	< 0.001	non-linear polynomial regression 4^th^ degree
**35–39 years**	0.070	< 0.001	non-linear polynomial regression 4^th^ degree
**40–44 years**	0.097	< 0.001	non-linear polynomial regression 5^th^ degree
**45–49 years**	0.082	< 0.001	non-linear polynomial regression 4^th^ degree
**50–54 years**	0.069	< 0.001	linear
**55–59 years**	0.068	< 0.001	linear
**60–64 years**	0.051	< 0.001	linear
**65–69 years**	0.087	< 0.001	linear

The trend of the regression (linear or non-linear) is also inserted.

**Table 2:** **Running speed of the annual ten fastest at the start and at the end of the investigated period for women and men in 100 km and 100 miles**

	Running speed of the annual ten fastest at the start of the period (km/h)	Running speed of the annual ten fastest at the end of the period (2013) (km/h)	Percent change (%)
**100 km women**			
**25–29 years**	9.73 ± 2.32 (1985)	12.56 ± 0.18	29.1
**30–34 years**	8.40 ± 1.02 (1985)	12.60 ± 0.60	50.0
**35–39 years**	10.83 ± 0.99 (1985)	12.43 ± 0.27	14.7
**40–44 years**	10.20 ± 1.03 (1982)	12.21 ± 0.35	19.7
**45–49 years**	9.76 ± 1.26 (1984)	11.60 ± 0.19	18.8
**50–54 years**	9.84 ± 1.10 (1987)	10.85 ± 0.45	10.3
**55–59 years**	7.63 ± 1.66 (1989)	10.48 ± 0.49	37.3
**60–64 years**	9.25 ± 0.60 (1996)	9.71 ± 0.66	4.9
**100 km men**			
**18–24 years**	9.30 ± 0.65 (1971)	12.89 ± 1.20	38.6
**25–29 years**	9.56 ± 1.79 (1971)	14.25 ± 0.30	49.1
**30–34 years**	9.86 ± 0.96 (1971)	14.48 ± 0.23	46.8
**35–39 years**	10.93 ± 1.47 (1971)	14.56 ± 0.28	33.2
**40–44 years**	10.37 ± 1.29 (1971)	14.56 ± 0.25	40.4
**45–49 years**	9.93 ± 0.81 (1971)	13.73 ± 0.34	38.3
**50–54 years**	9.18 ± 0.81 (1971)	12.53 ± 0.16	36.5
**55–59 years**	8.92 ± 1.41 (1974)	11.82 ± 0.42	32.5
**60–64 years**	9.80 ± 0.53 (1985)	11.22 ± 0.29	14.5
**65–69 years**	7.35 ± 1.66 (1985)	10.75 ± 0.34	46.3
**70–74 years**	7.67 ± 0.90 (1991)	9.07 ± 0.70	18.3
**75–79 years**	5.21 ± 0.46 (1996)	7.99 ± 0.38	53.4
**100 miles women**			
**25–29 years**	6.65 ± 1.05 (2001)	8.58 ± 0.68	29.0
**30–34 years**	6.21 ± 0.57 (1995)	8.61 ± 0.52	38.6
**35–39 years**	7.07 ± 1.29 (1985)	9.00 ± .090	27.3
**40–44 years**	7.69 ± 1.56 (1983)	8.58 ± 0.28	11.6
**45–49 years**	6.77 ± 1.39 (1989)	9.58 ± 0.69	41.5
**50–54 years**	6.09 ± 1.16 (1996)	8.00 ± 0.23	31.4
**55–59 years**	6.03 ± 0.58 (2001)	6.40 ± 0.32	6.1
**100 miles men**			
**18–24 years**	6.38 ± 0.94 (1998)	8.51 ± 0.72	33.4
**25–29 years**	7.86 ± 1.02 (1979)	10.49 ± 1.16	33.5
**30–34 years**	7.98 ± 0.73 (1979)	10.46 ± 0.56	31.1
**35–39 years**	7.86 ± 0.55 (1979)	9.71 ± 3.54	23.5
**40–44 years**	7.62 ± 0.95 (1979)	11.32 ± 0.51	48.5
**45–49 years**	7.39 ± 0.93 (1979)	10.06 ± 0.75	36.1
**50–54 years**	5.98 ± 0.61 (1982)	9.67 ± 0.53	61.7
**55–59 years**	6.57 ± 0.77 (1986)	8.76 ± 0.49	33.3
**60–64 years**	6.45 ± 0.42 (1996)	8.24 ± 0.77	27.7
**65–69 years**	5.64 ± 0.21 (1996)	6.33 ± 0.51	12.2

In 100 miles in women, running speed increased in all age groups (Figure 4). The increase was linear in 25–29 (Figure 4A) and 30–34 years (Figure 4B), non-linear in 35–39 (Figure 4C), 40–44 (Figure 4D), and 45–49 years (Figure 4E), but again linear in 50–54 (Figure 4F) and 55–59 years (Figure 4G). The highest percent change was found in age group 45–49 years (Table 2). For men in 100 miles, running speed increased linearly in 18–24 years (Figure 5A), non-linearly in 25–29 (Figure 5B), 30–34 (Figure 5C), 35–39 (Figure 5D), 40–44 (Figure 5E), and 45–49 years (Figure 5F), but again linearly in 50–54 (Figure 5G), 55–59 (Figure 5H), 60–64 (Figure 5I) and 65–69 years (Figure 5J). The highest percent change was found in age group 50–54 years (Table 2).

## Discussion

This study found that athletes in younger to middle age groups (*i.e.* 25–35 to 50–65 years) depending upon sex and race distance seemed to have reached their limits due to a non-linear increase in running speed whereas runners in older age groups (*i.e.* older than 50–65 years) depending upon sex and race distance might still improve their performance due to a linear increase in running speed. However, very young ultra-marathoners (*i.e.* younger than 25–35 years) might also still be improving their performance. This might especially be true for female (*i.e.* age groups 25–29 and 30–34 years) and male (*i.e.* age group 18–24 years) 100 miles ultra-marathoners.

### Increase in participation for all age groups in women and men

A first important finding was that the increase in both female and male finishers in 100 km ultra-marathons was due to an exponential increase in female and male finishers of all age groups. Similar findings have been reported for 100 miles ultra-marathons held in North America. Participation and performance trends in 100 miles ultra-marathons held in the USA such as the ‘Western States 100-Mile Endurance Race’ have been investigated by Hoffman (2010) and Hoffman and Wegelin (2009). Between 1977 and 2008, a total of 32,352 finishes were achieved by 9,815 individuals (Hoffman et al. 2010b). The annual number of races and the annual number of finishes increased exponentially through a combination of an increase in participation of runners > 40 years and an increase in participation of women (Hoffman et al. 2010b). Participation among runners > 40 years of age increased from less than 40% of the finishes prior to the mid-1980s to 65–70% of the finishes since 1996. In women, participation increased from practically no female starter in the late 1970s to nearly 20% since 2004 (Hoffman et al. 2010b; Hoffman and Wegelin 2009).

Age has been reported to be an important predictor variable for 100 km ultra-marathon race times in men (Knechtle et al. 2010a). In recent decades, a continuous increase in the number of ‘older’ participants in sporting events such as running, swimming, cycling, rowing, and weightlifting has been reported (Trappe 2001). The aging society in the general population could provide a reason why the numbers of master runners increased. The number of older people in the general population increased in recent decades (Dini and Goldring 2008; Robine and Paccaud 2005; Robine et al. 2003; Savidan et al. 2010; Wanner 2002) and also the maximum life-span increased (Wilmoth et al. 2000). These increases might be responsible for the growing number of master marathoners (Lepers and Cattagni 2012) and master ultra-marathoners (Zingg et al. 2013a, [b]). As the share of older people increased over the years in the general population, a higher number of master runners might have the opportunity to participate in sports such as running competitions. For example, Van Gool et al. (2011) investigated in the Netherlands between 1990 and 2007 whether an increase in life expectancy was associated with a decrease in physical activity limitations. They found that even though life expectancy increased, limitations of physical activity did not decrease. The gain of more years in life seemed to influence the number of potential master runners but did not appear to influence their level of physical activity.

Another explanation could be the fact that it is inherent to ‘new’ sport events. If ‘new’ sport events persist for more than a couple of years the participation rates will increase and - as the age of the athletes increases - so will the participation rate in older age groups. However, this was not the case in the longest inline race in Europe, the ‘Inline 111’ (Teutsch et al. 2013) and one of the first ultra-distance mountain bike races in the world, the ‘Swiss Bike Masters’ (Gloor et al. 2013). The ‘Inline 111’ had to stop after a few years of prosperity (Teutsch et al. 2013) and the ‘Swiss Bike Masters’ (Gloor et al. 2013) had to reduce the length of the race distance due to a decrease in participants most probably due to other mountain bike races held in the Alps.

### Improved performance in 100 km and 100 miles ultra-marathoners

A second important finding was that athletes in all age groups improved performance with the exception of female 100 km ultra-marathoners in age group 25–29 years. There was a non-linear increase in running speed in female 100 km ultra-marathoners in age groups 30–34 to 60–64 years, in male 100 km ultra-marathoners in age groups 18–24 to 60–64 years, in female 100 miles ultra-marathoners in age groups 35–39 to 45–49 years and in male 100 miles ultra-marathoners in age groups 25–29 to 45–49 years. In contrast, a linear increase in running speed was found for female 100 km ultra-marathoners in age group 60–64 years, for male 100 km ultra-marathoners in age groups 65–69 to 75–79 years, in female 100 miles ultra-marathoners in age groups 25–29, 30–34, 50–54 and 55–59 years and in male 100 miles ultra-marathoners in age groups 18–24, and 50–54 to 65–69 years. A non-linear increase in running speed suggests that athletes in these age groups seemed to have reached their limits in running performance. In 100 km races, more age groups showed a non-linear increase in running speed than in 100 miles races. Therefore, 100 km ultra-marathoners seemed rather to have reached their limits in running speed compared to 100 miles ultra-marathoners. On the other side, 100 miles age group ultra-marathoners seemed to have still the possibility to improve their performance in the future due to the linear increase in running speed. This seems especially to be true for younger age group athletes (*i.e.* 18–24 years in men and 25–34 years in women) and older age group athletes (*i.e.* older than 50 years for both women and men).

Potential explanations for these disparate findings could be the longer race time in 100 miles (*i.e.* 161 km) ultra-marathons (Hoffman and Wegelin 2009) compared to 100 km ultra-marathons (Knechtle et al. 2012b) and differences in anthropometry and physiology between female and male ultra-marathoners (Hoffman 2008). The fastest 100 km race times are 6:13:33 h:min:sec for men, set in 1998 by Takahiro Sunada, and 6:33:11 h:min:sec for women, set in 2000 by Tomoe Abe (IAAF Athletics, http://www.iaaf.org). For 100 miles, the fastest race times in road running was achieved for men by Andy Jones in 1997 with 12:05:43 h:min:sec and for women by Ann Trason in 1991 with 13:47:41 h:min:sec (44). The 100 miles are 61 km longer than the 100 km (61%), the difference in the world’s best times between 100 km and 100 miles are 347 min for men (47.9%) and 434 min for women (52.5%). The difference between the fastest 100 km race times for women and men was 20 min (4%), but 102 min (14.1%) for the 100 miles race times. In other terms, the 61 km longer race distance in the 100 miles increased the sex difference in performance in the world’s fastest race time by 10%.

Regarding anthropometric characteristics, it has been shown that female ultra-marathoners have a lower skeletal muscle mass and a higher percent body fat (Knechtle et al. 2010b) compared to male ultra-marathoners (Knechtle et al. 2011d). Since low body fat has been reported to be associated with faster ultra-marathon race times (Rüst et al. 2012) and ultra-marathon running is associated with a decrease in skeletal muscle mass (Knechtle et al. 2012a), men seemed to have an advantage for the 100 miles distance as the longer ultra-marathon distance compared to the 100 km distance.

Another explanation could be methodical aspect that the annual top ten athletes for each age group were analysed. Regarding the 100 km races, ten and more men were already in 1971 considered for nearly all age groups. For women in 100 km races, however, for the first time at least ten women finished in 1982 in age group 40–44 years. It becomes even more obvious for 100 miles races where in 1983 for the first time at least ten women finished in any age group (35–39 years) and in 1979 where at least ten men finished in age groups 25–29 to 45–49 years. The later appearance of ten and more age group finishers in 100 miles ultra-marathons might explain that more age group athletes showed a linear increase in running speed in 100 miles. The inclusion of a fixed number such as the annual top twenty (Lepers and Cattagni 2012) or annual top fifty (Jokl et al. 2004) for each age group in marathoners or the annual top ten for each age group in ultra-marathoners (Knechtle et al. 2012b) might lead to a selection bias. An analysis with the inclusion of all recorded finishers for each age group might omit a potential selection bias and may lead to different results.

It is important to mention that participation in master ultra-marathoners increased and they were able to improve their performance although it is known that more master runners become injured than younger runners and more master runners suffered multiple injuries than younger runners (McKean et al. 2006) which might limit their performance. The prevalence of soft-tissue-type injuries to the calf, Achilles tendon, and hamstrings was greater in master runners than in younger runners whereas younger runners suffered more knee and leg injuries than masters runners (McKean et al. 2006). Although running more times per week increased the risk of injury for both younger and master runners (McKean et al. 2006) and overuse injuries of the lower limbs became more common in master runners (Fields 2011), master runners were able to improve performance across years. This was most probably due to differences in training (*e.g.* training intensities) between younger and master runners (McKean et al. 2006). Master runners may have increased running volume and running intensity. Indeed, a training distance < 40 km a week was predictive for future calf injuries and regular interval training was predictive for future knee injuries (Van Middelkoop et al. 2008).

### Strengths, limitations and implications for future research

The strength of this worldwide study is the large data set including all finishers in 100 km and 100 mile ultra-marathons held between 1971 and 2013 and the comparison between linear and non-linear regressions. However, some races might not have been recorded in http://www.ultra-marathon.org but the largest 100 km and 100 miles ultra-marathons were all listed. This cross-sectional data analysis suffers some limitations since variables such as training (Knechtle et al. 2012a), anthropometric characteristics (Hoffman 2008; Rüst et al. 2012; Gianoli et al. 2012; Hoffman et al. 2010a), previous experience (Knechtle et al. 2010c; Knechtle et al. 2011a, [b], [c]), motivation (Hodge et al. 2008; Krouse et al. 2011; Ruiz-Juan and Zarauz 2012), demographic characteristics (Hoffman and Fogard 2012), nationality (Cejka et al. 2014) and performance-limiting factors (Hoffman and Fogard 2011) were not included. The selection of the annual top ten for each age group might lead to a selection bias due to low numbers of finisher in the early years of ultra-marathon running. Future studies might include all recorded finishers for each age group.

## Conclusions

The number of successful finishers increased exponentially in all age groups in 100 km and 100 miles ultra-marathons in both women and men. Athletes in younger to middle age groups (*i.e.* 25–35 to 50–65 years depending upon sex and distance) seemed to have reached their limits in performance due to a non-linear increase in running speed whereas runners in very young (*i.e.* younger than 25–35 years) and older age groups (*i.e.* older than 50–65 years depending upon sex and distance) might still improve their performance due to a linear increased in running speed. In 100 miles, especially younger athletes (*i.e.* 18–24 years in men and 25–34 years in women) and older athletes (*i.e.* older than 50 years for both women and men) might still have the possibility to improve their performance in the near future. Overall, the faster race times over the last 30 years are a result of all top ten finishers getting faster.
